# Bioavailability of iodine from a meal consisting of sushi and a wakame seaweed salad—A randomized crossover trial

**DOI:** 10.1002/fsn3.3689

**Published:** 2023-09-24

**Authors:** Inger Aakre, Inger Beate Tveit, Lene Secher Myrmel, Even Fjære, Simon Ballance, Hanne Rosendal‐Riise

**Affiliations:** ^1^ Marine Toxicology Institute of Marine Research Bergen Norway; ^2^ Department of Clinical Medicine University of Bergen Bergen Norway; ^3^ Feed and Nutrition Institute of Marine Research Bergen Norway; ^4^ Nofima AS Norwegian Institute of Food, Fisheries and Aquaculture Research Ås Norway; ^5^ Mohn Nutrition Research Laboratory and Center for Nutrition, Department of Clinical Medicine University of Bergen Bergen Norway

**Keywords:** bioavailability, bioequivalence, iodine, macroalgae, seaweed, sushi

## Abstract

The consumption of seaweed is on the rise in the Western world. Seaweeds may contain substantial amounts of iodine, and some species could serve as a potential dietary iodine source. However, limited data on the iodine content and in vivo bioavailability of iodine from seaweeds exist. The objective was to assess whether iodine from a meal consisting of sushi with nori, (*Porphyra* spp) and a wakame seaweed salad (*Undaria pinnatifida*) had similar bioavailability as a potassium iodide reference supplement of similar iodine content. A randomized 2 × 2 crossover trial (AB/BA model) was conducted in 20 healthy young women. One intervention arm consisted of a meal with sushi and wakame salad (231 μg iodine), and the other of potassium iodide (KI) supplement (225 μg iodine). Urinary iodine concentration (UIC) was measured at 11 different time points for 48 h after the interventions. The UIC increased after consumption of both the sushi and wakame meal and the KI supplement, but the median UIC was higher after ingestion of the KI supplement. The estimated bioavailability of iodine during the first 24 h was 75% from sushi with wakame and 97% from the KI supplement. The bioequivalence analyses confirmed that the KI supplement had higher estimated bioavailability than the sushi and wakame meal, however, with small margins. Our findings on iodine bioavailability imply that sushi and wakame could be potential iodine sources in the diet, which may be favorable for population groups at risk for iodine deficiency. However, further research is needed to account for the variability of iodine content in seaweeds by different locations and degree of processing, to assure that the iodine levels are stable and predictable for the consumers.

## Graphical abstract

The objective was to assess whether iodine from a meal consisting of sushi with nori (*Porphyra* spp) and a wakame seaweed salad (*Undaria pinnatifida*) had similar bioavailability as a potassium iodide reference supplement of similar iodine content. A randomized 2 × 2 crossover trial (AB/BA model) was conducted in 20 healthy young women. Urinary iodine concentration increased after consumption of both the sushi and wakame meal and the KI supplement but was higher after ingestion of the KI supplement.

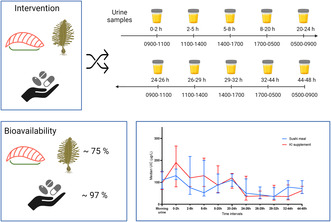

## 1 INTRODUCTION

Iodine is an essential trace element for humans. It is a component of the thyroid hormones triiodothyronine (T3) and thyroxine (T4) which have important roles in metabolic regulation, neurological development, and growth (Brent, 2012). Both inadequate and excess iodine intake may alter thyroid function, which increases the risk of adverse health consequences (Laurberg et al., 2009). The health consequences of severe iodine deficiency are well documented and are often referred to as iodine deficiency disorders (IDDs). Mild‐to‐moderate iodine deficiency may result in less severe outcomes than severe deficiency, where studies have found associations between mild iodine deficiency during pregnancy and reduced fetal growth (Abel et al., 2020) and impaired neurodevelopment (Abel et al., 2017). Altered thyroid function has also been associated with excessive iodine intake in several population groups (Katagiri et al., 2017). There is, however, a limited number of studies regarding the consequences of mild‐to‐moderate iodine deficiency and iodine excess and the evidence is not conclusive.

Despite great progress has been made in eliminating iodine deficiency, it is still one of the most common nutritional disorders worldwide (Iodine Global Network (IGN), 2021). In vulnerable groups, such as pregnant women, the IGN reported that 54% of the world's countries had insufficient iodine status. Europe was the region with the highest prevalence, where 75% of the countries reported iodine deficiency among pregnant women (Gizak et al., 2017; IGN, 2017). Mild‐to‐moderate iodine deficiency has been documented in several population groups in Norway, such as young (Henjum et al., 2018), pregnant, and lactating women (Aakre, Morseth, et al., 2021; Abel et al., 2018; Henjum et al., 2017).

Iodine is not stored in large quantities in the body, and regular consumption is necessary (Zimmermann, 2009), by including dietary iodine sources as a part of the everyday diet. Norway is a country without mandatory salt iodization; therefore, iodine must be consumed through foods (Dahl et al., 2004). The greatest iodine reservoir in nature is the ocean (Mehra et al., 2014). Marine foods are, therefore, good iodine sources, and the highest content is found in white fish, shellfish, and seaweed (Aakre, Doblaug Solli, et al., 2021; Nerhus et al., 2018). In Norway, milk and dairy products constitute the most important iodine source since animal feed is enriched with iodine, and milk and dairy are consumed more frequently than lean fish (Nerhus et al., 2018). However, dairy intake has decreased in some population groups, making them more prone to iodine deficiency (The Norwegian Directorate of Health, 2021). Furthermore, plant‐based diets are increasing, and a study of Norwegian vegetarians and vegans found a median urinary iodine concentration (UIC) of 67 and 43 μg/L in the two groups, respectively, indicating an insufficient iodine intake (Groufh‐Jacobsen et al., 2020). Thus, alternative iodine sources are of interest.

Seaweeds, also called macroalgae, maybe a dietary iodine source, especially for those who are omitting animal‐based foods. Macroalgae are divided into three taxonomic groups based on pigmentation: brown (*Ochrophyta*), green (*Chlorophyta*), and red macroalgae (*Rhodophyta*) (Mæhre et al., 2014). Nutrient and iodine content varies both between and within the different groups and distinct species of macroalgae. Iodine content is generally higher in brown macroalgae compared to red and green macroalgae (Duinker et al., 2020). It may range from 16 μg/g in nori, a red macroalga, to over 6000 μg/g in sugar kelp (*Saccharina latissima*), one of the most commonly farmed brown macroalga in Norway (Blikra et al., 2022; Teas et al., 2004). Seaweed is becoming increasingly popular in the Western part of the world, especially sushi wrapped in nori and seaweed salad, also called wakame. There are, however, limited data on the iodine content of different seaweed products in the Norwegian Food Composition Table. Furthermore, there is limited research regarding in vivo bioavailability of iodine from seaweeds. It has been suggested that the bioavailability of iodine from seaweeds is low due to iodine being organically bound to different substances in the seaweed food matrix, such as proteins, pigments, polyphenols, and polysaccharides (Cherry et al., 2019; Combet et al., 2014). Additionally, high amounts of alginate in brown seaweeds have been suggested to trap iodide in viscoelastic gels and form insoluble compounds that are excreted in feces (Aquaron et al., 2002). Studies of bioavailability are necessary to understand how readily iodine from seaweeds is absorbed during digestion. Furthermore, the European Food Safety Authorities warrants such data to give advice regarding safe intake of macroalgae among consumers (EFSA et al., 2023).

The objective of this study is to assess whether iodine from a meal consisting of sushi including nori (*Porphyra* spp) and a wakame salad (*Undaria pinnatifida*) has similar bioavailability as a potassium iodide reference supplement of similar iodine content.

## 2 METHODS

This study was designed as a randomized 2 × 2 crossover trial (AB/BA model) where participants enrolled were randomized to either the sushi and wakame meal or an iodine supplement in the first intervention. All participants had at least 7‐day washout period between interventions. The study was conducted at Research Unit for Health Surveys (RUHS), in Bergen, Norway.

### 2.1 Participants

The study population was recruited as a subsample from a larger comparison study (Rosendahl‐Riise et al., 2023), including young healthy women aged 18–40 years. All participants entering the comparison study were informed about the crossover trial and allowed to participate if the inclusion criteria were met. Exclusion criteria in the current study were as follows: pregnant or lactating, known thyroid disease or thyroid autoimmunity, planning to conceive within the study period, known kidney problems, or kidney disease. In addition, women with coeliac disease could not participate due to possible traces of gluten in the wakame salad.

Sample size and power calculations were carried out in R statistical software using the (Power.TOST) package employing Owen's Q exact method for bioequivalence studies. Log‐transformed data (multiplicative model), alpha (significance level) = .05, target power = 0.8, assumed T/R ratio = 0.95, geometric coefficient of variation = 0.2, and standard bioequivalence margins of 0.8–1.25 were used for “AUClast” as the primary outcome (EFSA et al., 2021). A sample size of *n* = 20 was found to have a power of 83%. Due to possible loss to follow‐up, two extra participants were added, giving a total sample size of 22 for the study.

### 2.2 Interventions

The interventions were a meal with sushi and wakame salad, and the comparator was an iodine supplement. Sushi and wakame salad were chosen as the intervention meal as they are popular products often consumed together, and they are commercially available in many food stores. The sushi and wakame meal were bought at two local supermarkets in Norway. Macroalgae species in the meal were wakame (*Undaria pinnatifida*), which is a brown macroalga, and nori (*Porphyra* spp), which is a red macroalga. The sushi and wakame meal consisted of 10 sushi pieces: six maki rolls and four nigiri with salmon. The maki rolls consisted of salmon (2 pieces), surimi (2 pieces), and shrimp (2 pieces) with nori sheets, and the total weight of the sushi was 229 g, excluding soy sauce, wasabi, and ginger. The wakame salad was 80 g per serving, and marinated in a soy sauce mixture. The sushi and wakame were served together in the intervention meal. The iodine supplement was chosen based on being the only pure dietary iodine supplement available in Norwegian pharmacies. The declared iodine content was 225 μg per tablet by the manufacturer (Nycoplus), in the form of potassium iodide (KI). The dose of the intervention was one tablet. Participants were randomized to receive either the sushi and wakame meal or the iodine supplement in their first intervention. The order of the intervention (sushi and wakame or supplement) was unknown to the participants before the study visit.

### 2.3 Study procedure

**FIGURE 1:**
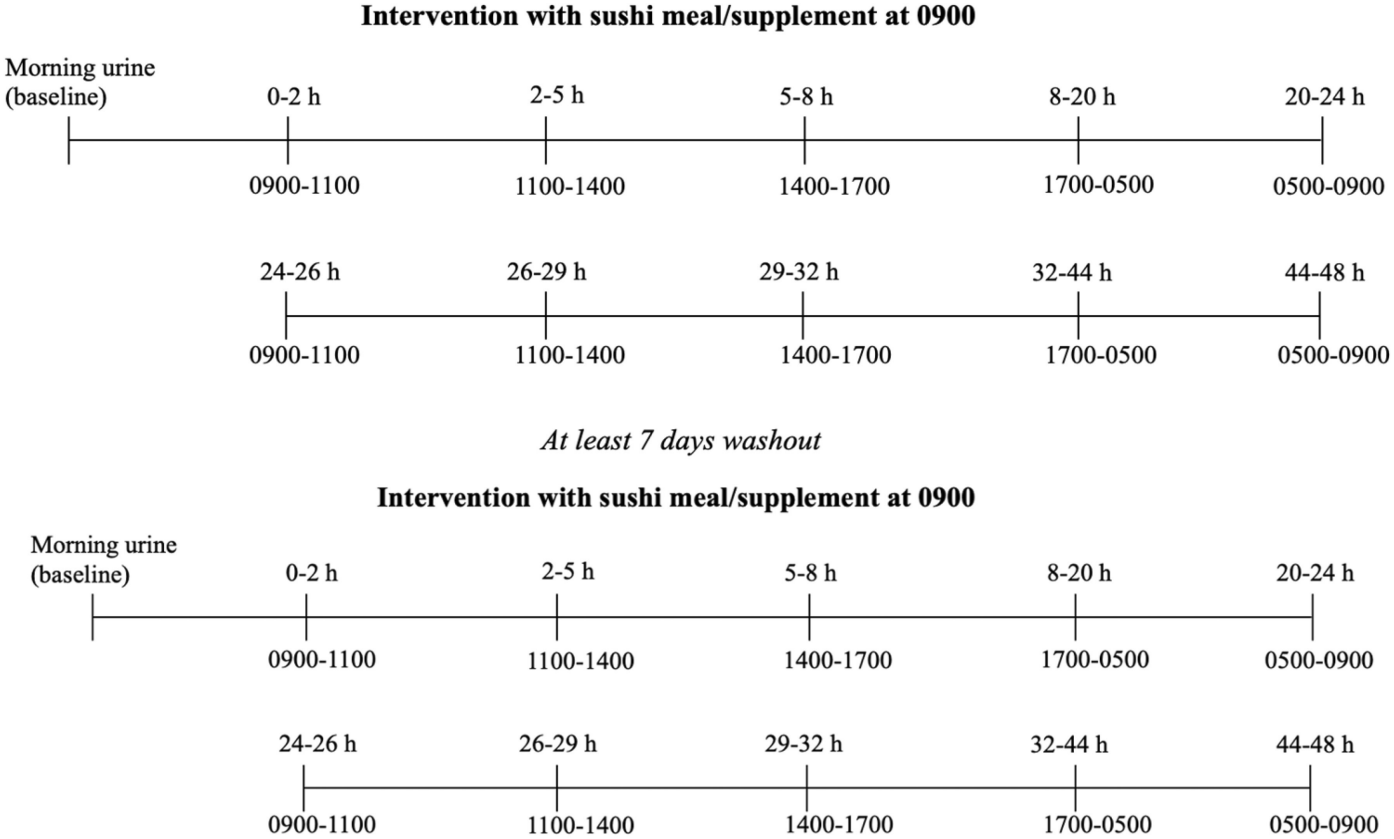
Overview of the schedule of spot urine samples given in hours post‐intervention and time intervals.

On the day of the intervention, the participants first took a morning spot urine sample at home before the study visit. At 9:00 a.m., they were provided with the intervention. After consuming the sushi and wakame meal or supplement, the participants were instructed to collect spot urine samples over 48 h in specific time intervals (Figure 1): 9 a.m.–11 a.m., 11 a.m.–2 p.m., 2 p.m.–5 p.m., 5 p.m.–5 a.m., 5 a.m.– 9 a.m., 9 a.m.–11 a.m., 11 a.m.–2 p.m., 2 p.m.–5 p.m., 5 p.m.–5 a.m., and 5 a.m.–9 a.m. Each study arm required 11 urine samples per person, in total 22 for both interventions. The second study visit was scheduled after a washout period of at least 7 days.

During the study visit and urine collection period, participants were asked to avoid iodine‐rich foods like milk and dairy products, white fish and fish products, eggs, seaweed, and supplements containing iodine to minimize interference with other iodine‐rich foods. The participants were also asked to register their diet in the first intervention, and to repeat it during the second intervention. The purpose was to make the conditions as similar as possible. At the end of each intervention arm, the participants answered questions regarding compliance with the dietary restrictions. Iodine intake from the self‐reported deviations from protocol was calculated by using the digital diet tool from the Norwegian Directorate of Health and the Norwegian Food Safety Authority based on data from the Food Composition Table version 2022 (Norwegian Directorate of Health & Norwegian Food Safety Authority, 2022).

### 2.4 Urinary iodine concentration

Spot urine samples were kept refrigerated (0–4°C) before they were handed over to study personnel. Each sample was homogenized before extracting 10 mL using an automatic Sarstedt pipette, transferred to Sarstedt tubes, and stored at −20°C pending analyses.

Analyses of iodine concentration were done at the Institute of Marine Research (IMR), by ICP‐MS with autosampler Agilent ASX‐500. For analysis of iodine concentration, 500 μL urine was diluted in 4.5 mL of tetramethylammonium hydroxide (TMAH) with a concentration of 1%, following filtering through a sterile membrane with a pore size of 0.45 μm and a single‐use syringe (Julshamn et al., 2001). Specimens were analyzed against a standard addition curve (urine calibration curve) to quantify the unknown iodine content in the urine samples. Internal validity of the method was verified with certified reference material; Seronorm Trace Elements Urine. For the whole measurement range, measurement uncertainty for iodine is 20%. The limit of quantification (LOQ) for iodine concentration in the urine samples was 7.8 μg/L.

### 2.5 Sampling of the sushi and wakame meal for analyses of iodine content

For the determination of iodine content in the sushi and wakame meal, three products from three different batch numbers of sushi and wakame were selected. If different batch numbers were not accessible, different expiration dates were used. Three sushi trays from the same batch number/expiration date were pooled into a composite sample, giving a total of three composite samples of sushi. Ginger, wasabi, and soy sauce were not included in the analysis of iodine content. Similarly, three composite samples of wakame salad were made.

Analyses of the sushi and wakame samples were conducted at IMR by ICP‐MS with High Matrix Introduction and autosampler PrepFast. Samples were homogenized, freeze‐dried, and re‐homogenized. Then, 0.2 g of material was transferred to 50 mL centrifugation tubes, and 5 mL of MilliQ distilled water and 1 mL of concentrated TMAH were added. Samples were put in 90°C heating cabinets for 3 h, before cooling and dilution to 25 mL with MilliQ distilled water, following filtering through a sterile membrane with a pore size of 0.45 μm and a single‐use syringe, before determining the iodine content by ICP‐MS.

### 2.6 Estimation of bioavailability

Area under the curve (AUC) for UIC values in the intervention groups were calculated in Excel, by the trapezoidal method. Zero was used as the baseline input. Calculations have been made for the whole intervention period, as well as for each 24‐h period. Morning spot urine samples were not included in the calculations. Since the samples were taken in intervals, the end of each interval was used as input in the calculations, starting at 2 h and ending at 48 h, giving a total of 10 interval areas. The AUC for UIC was used to assess bioequivalence between the iodine supplement and test meals according to standard statistical methods (EFSA et al., 2021; appendix D). Bioequivalence is a term used in pharmacology to describe the relative efficacy of two different formulations of a drug. Bioequivalence methods were applied in this study to evaluate bioavailability of the sushi and wakame meal.

An estimated bioavailability was calculated for the first 24 h based on an estimated urinary iodine excretion (UIE), using a urine volume of 1.5 L (IOM, 2001; Zimmermann, 2012), by the following equation: UIE = mean UIC (μg/L) × 1.5 (L). Subsequently, the estimated amount of iodine excreted was compared to the analyzed and declared iodine content in the interventions. This provided an estimate of how much percent was excreted from the amount consumed. The morning urine baseline sample was excluded from the calculations. The following time points were included in the first 24 h: 0–2 h, 2–5 h, 5–8 h, 8–20 h, and 20–24 h.

### 2.7 Questionnaire

The participants filled in a questionnaire regarding background information including age, weight, height, country of birth, years living in Norway, education, smoking, seaweed consumption, and dietary patterns. Regarding seaweed consumption, participants were asked if they had a habitual dietary intake of whole‐food algae products (i.e., sea spaghetti, seaweed salad, kombu, wakame, dried seaweeds, etc.), foods containing seaweed (i.e., sushi with nori, seaweed seasoning blends, tare oil, pastry with seaweeds, etc.), or supplements containing algae or seaweed. If yes, frequency of intake was requested. The frequencies were as follows: “daily,” “4–6 times per week,” “1–3 times per week,” “less frequently than weekly,” and “other (specify).”

Data on UIC before the start of this study was obtained from the comparison study from a pooled sample of six spot urine samples taken over 6 consecutive days, to assess the participants' iodine status before the intervention. These samples were analyzed at the IMR according to the methods described earlier. In addition, TSH, fT3, fT4, TPOAb, Tg, and TgAb were measured to assess the thyroid function of the participants before the intervention. Venous serum sampling was performed by bioengineers at RUHS using butterfly cannulas, collected in 5 mL BD Vacutainer tubes with gel. Blood samples were set to coagulate for 30 min, following centrifugation in Eppendorf 5702 at 2000G for 10 min at room temperature. 0.5 mL aliquots of serum were extracted from the blood sample tube and transferred to Sarstedt tubes, three aliquots per participant. The serum samples were then transiently stored at −80°C freezers at RUHS. Serum samples were analyzed by electrochemiluminescence immunoassay (ECLIA) at Haukeland University Hospital (certified laboratory NS‐EN‐ISO15189).

### 2.8 Ethics

The present study was approved by the Regional Committee for Medical and Health Research Ethics in Norway (reference number 232247). Participants were provided with information about the study both orally and in writing and had to sign a consent form in order to be enrolled in the study. Enrolled participants could withdraw at any time without giving a reason. The trial was registered in ClinicalTrials.gov, NCT05773456.

### 2.9 Data management and statistics

Data management and statistical analyses were conducted in Excel version 2210, Statistical Package for Social Sciences version 27, IBM Corporation (SPSS), and R. Descriptive statistics on background characteristics and iodine status were presented as medians with 25p–75p for continuous variables, and count (*n*) and frequency (%) for categorical variables. Normality was assessed by visual inspection of normality plots. For the iodine content analyses of the sushi meal and wakame salad, results were presented as average iodine content (μg) per 100 g of pooled samples (wet weight). Values below LOQ (*n* = 3 for UIC) were set to the detected at LOQ value of 7.8 μg/L. Results for UIC are given as medians and 25p–75p due to the non‐normal distributions. The following results were presented and estimated using means and standard deviations: estimated iodine intake from self‐reported deviations from protocol, and estimated bioavailability. A statistical assessment of bioequivalence between the iodine supplement and test meals was made according to standard methods (EFSA et al., 2021; appendix D). The package “BE” for bioequivalence testing in R statistical software was employed.

## 3 RESULTS

In total, 22 young and healthy women were included in this randomized crossover trial. One participant withdrew during the study, after finishing the first study visit, and was excluded from the statistical analyses. One participant who did not finish the wakame salad, leaving behind 55 g, was also excluded from the analysis, giving a final sample size of 20 participants in the statistical analyses. In the crossover trial, 40% of the participants were randomized to start with the sushi meal intervention, while 60% were randomized to start with the KI supplement. No adverse effects were reported during the interventions. All participants but one finished the wakame salad, leaving behind 5 g. As for the sushi, 14 participants finished the meal while six participants did not. The amounts not eaten were as follows: two maki rolls (*n* = 1), one nigiri (*n* = 2), two nigiri (*n* = 2), and three nigiri (*n* = 1). Regarding the KI supplement, all participants (*n* = 20) ingested the tablet. Self‐reported deviations from the dietary restrictions were under 10 μg in both study arms (Table S1). All urine samples at all timepoints, except one that was forgotten, were collected by the participants.

Background variables are described in Table 1 and iodine status as well as thyroid function tests before the intervention are given in Table 2. The median (25p–75p) age of the participants was 27 (24–31) years, and the majority had more than 4 years of higher education. Most of the participants studied or worked within health sciences. None of the participants were vegetarian or vegan (data not shown). The median (p25–p75) UIC prior to the intervention was 79 (63–113) μg/L. None of the participants had biochemically assessed thyroid dysfunction, but three participants were TPOAb and TgAb positive.

**TABLE 1:** Background characteristics in the young women included in the randomized cross‐over trial (*n* = 20).

	Median	25p–75p
Age (years)	27	24–30
Body weight, self‐reported (kg)	66	59–74
Height, self‐reported (cm)	169	165–173
BMI (kg/m^2^)	23	21–25

	*n*	Percent
Education status		
13 years of high school	3	15
1–4 years of college/university	4	20
4+ years of college/university	13	65
Marital status		
In a relationship	6	30
Cohabitant	6	30
Single	8	40
Daily tobacco use^a^	1	5
Thyroid disease (self‐reported)	None	0
Medication for thyroid disease	None	0

^a^ Nonsmoke tobacco.

**TABLE 2:** Urinary iodine concentration (UIC) and thyroid function tests prior to the intervention (*n* = 20).

Biomarkers	Mean	Min, max	p10	p25	p50	p75	p90	Reference range^c^
UIC (μg/L)	88	27, 200	50	58	75	105	167	>100
s‐TSH (mIU/L)	1.6	0.7, 3.5	0.9	1.2	1.4	2.0	2.5	0.4–4.5
s‐fT4 (pmol/L)	15.8	12.5, 19.8	12.8	14.2	15.7	16.8	19.0	9.5–22.0
s‐fT3 (pmol/L)	4.9	3.3, 6.1	3.8	4.2	5.2	5.7	5.8	3.1–6.8
s‐Tg (μg/L)^a^ ^,^ ^b^	19.4	5.5, 42.0	6.3	12.3	18.5	23.3	38.4	<77.0

^a^ Two participants: Positive s‐TgAb and s‐TPOAb values.

^b^ s‐Tg values of participants with positive s‐TgAb were excluded (*n* = 18).

^c^ Reference range for UIC is based on the epidemiological criteria for assessing iodine nutrition from WHO, and the cut‐off of >100 is considered adequate (WHO, 2007). Reference ranges from the laboratory were used for thyroid function tests (Helse Bergen (Laboratorieklinikken), 2022a, 2022b).

The iodine content of the sushi and wakame meal is given in wet weight (Table 3). The mean ± SD iodine content in the wakame salad was 230 ± 36 μg/100 g and 20 ± 2.3 μg/100 g in the sushi. Per serving, mean iodine content was 47 ± 5.3 μg in sushi and 184 ± 28.8 μg in wakame, and in total, 231 μg iodine for the whole meal. The iodine content of the KI supplement was 225 μg, as declared by the manufacturer.

**TABLE 3:** Analyzed iodine content in the sushi and wakame salad, and declared iodine content in the KI supplement used in the randomized cross‐over trial.

Sample^a^	Iodine (μg/g) ww^b^	Iodine (μg/100 g) ww^b^	Serving size (g)	Iodine (μg) per serving
Sushi batch 1	0.19	19	229	43
Sushi batch 2	0.19	19	229	43
Sushi batch 3	0.23	23	229	53
Average ± SD	0.20 ± 0.02	20 ± 2.3	229	47 ± 5.3
Wakame salad batch 1	2.60	260	80	208
Wakame salad batch 2	2.40	240	80	192
Wakame salad batch 3	1.90	190	80	152
Average	2.30 ± 0.4	230 ± 36.1	80	184 ± 28.8
Total meal				231
Iodine supplement			1 tablet	225^c^

^a^ Each batch consists of three samples.

^b^ Wet weight.

^c^ Declared by manufacturer; not analyzed value.

The median (p25–p75) UIC from a pooled sample of six spot urine samples taken from six consecutive days prior to the intervention in the group randomized to start with the sushi and wakame meal (*n* = 8) were 89 (56–151) μg/L and 72 (59–103) μg/L in the group randomized to start with the supplement (*n* = 12). No statistically significant difference in UIC prior to the intervention period was found between these groups (*p* = .521). The baseline median UIC (25p–75p) in the morning spot urine samples was 90 (45–173) μg/L in the participants randomized to start with sushi and wakame (*n* = 8) and 115 (99–148) μg/L in the participants randomized to start with KI supplement ingestion (*n* = 12). There were no statistically significant differences in the morning urine samples (*p* = .571) between the two groups (data not shown). Median UIC in both groups was highest during the first 24 h (Table 4).

**TABLE 4:** Median UIC values for each 24‐h period after ingestion of a sushi meal with wakame salad and a KI supplement.

Intervention	*n*	UIC (μg/L) 0–24 h^b^		UIC (μg/L) 24–48‐h^c^		UIC (μg/L) 0–48‐h	
		Median (p25–p75)	Mean (min, max)	Median (p25–p75)	Mean (min, max)	Median (p25–p75)	Mean (min, max)
Sushi and wakame	20	105 (52–160)	115 (14, 330)	56 (29–95)	68 (7.8, 240)	76 (41–130)	91 (7.8, 330)
KI supplement	20^a^	120 (73–200)	145 (11, 440)	47 (23–87)	58 (8200)	81 (39–130)	101 (7.8, 440)

^a^ One sample missing from one participant in the 0–24 h interval.

^b^ Baseline sample (morning urine) excluded; samples included for each individual in the 0–24 h period is 5 (total no. of samples = 100 for each intervention).

^c^ Samples included for each individual in the 24–48 h period is 5 (total no. of samples = 100 for each intervention) Three UIC measures were < LOQ in the 24–48 h period for KI supplement and set to the LOQ value (7.8 μg/L) in the data analyses.

**FIGURE 2:**
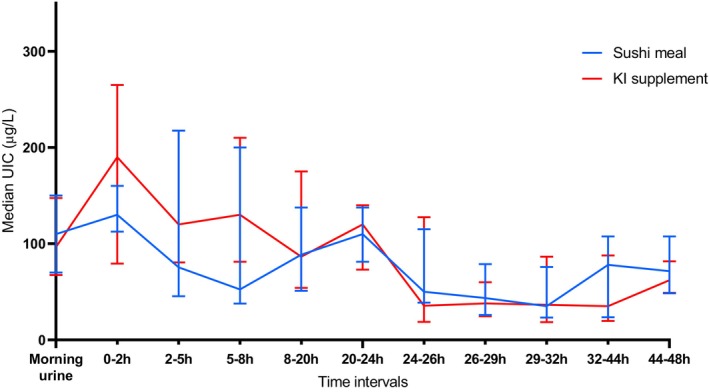
Median urinary iodine concentrations (μg/L) on group level over 48 h after ingestion of a sushi meal with wakame, and a KI supplement. Results are presented as the median for 20 participants in the sushi meal intervention group and 20 participants in the KI supplement intervention group. The bars represent p25–p75 for each time interval.

Median UIC (in μg/L per time interval) is shown over 48 h after consumption of a sushi and wakame meal and a KI supplement on group level in Figure 2. The visual trends show that the UIC increases within the early time intervals for both the supplement and sushi groups and that the increase seems higher in the supplement group. Regarding the spread of the data, there is greater variability in both groups during the first 24 h (Figure S1). There was an outlier in the KI supplement group, as shown in Figure S1.

The geometric mean for AUC of UIC in the whole intervention period, excluding the baseline urine samples, was 3946 (ln μg/h/L) in the KI supplement group compared to 3555 (ln μg/h/L) in the sushi and wakame group. When assessing similarity of the estimated bioavailability between the KI supplement (reference) and the sushi meal (test) (Table 5 and Appendix S1), the bioavailability was higher for the KI supplement in the 0‐ to 24‐h time period than for the sushi meal. During the next 24–48 h, the bioavailability was higher for the sushi and wakame meal, while for the whole time period, the bioavailability of iodine from the sushi and wakame meal was lower compared with the supplement. The maximum observed concentration (C_max_) for UIC was higher in the supplement group than in the sushi and wakame groups. The highest concentrations were found most frequently in the early time intervals for both intervention groups (0–2 and 5–8 h) (data not shown).

**TABLE 5:** Assessment of similarity in bioavailability of iodine from a sushi and wakame meal and a supplement at different time points.^a^

Parameter	Reference (R) or test (T)	Geometric mean	90% confidence interval of geometric mean ratio (T/R)^b^		Bioavailability of T *higher* or *lower* than R
		(ln μg/h/L)	Lower CI limit	Upper CI limit	
AUC_0–24_	R	2832	0.64	0.96	
	T	2235			Lower
AUC_24–48_	R	1016	1.02	1.44	
	T	1235			Higher
AUC_0–48_	R	3946	0.78	1.04	
	T	3555			Lower

		**(ln μg/L)**			
Cmax_0–48_	R	214	0.63	0.99	
	T	169			Lower

^a^ Assessment of similarity in bioavailability is based on 90% confidence intervals for the ratio of the geometric means (test/reference) of AUC for first the 24 h, last 24 h, and 48 h and for maximum urinary iodine concentration observed over 48 h.

^b^ CI limit below 0.8 suggests iodine bioavailability in the sushi and wakame meal (test (T)) is less than in the iodine supplement (reference (R)). A CI limit above 1.25 suggests iodine bioavailability in the sushi meal (test (T)) is greater than in the iodine supplement (reference (R)).

The estimated bioavailability of iodine based on estimated UIE during the first 24 h was higher after ingestion of a KI supplement (97%) compared to ingestion of sushi and wakame (75%).

We assessed habitual macroalgae consumption among the participants in the study. One participant included whole‐food macroalgae (dried sugar kelp, winged kelp, and dulse) in the diet weekly, while 15 participants included foods with macroalgae as an ingredient in their diet. Of these, all consumed sushi with nori sheets (14 less than weekly and 1 weekly), while four participants also used seaweed seasoning blends such as seaweed salt monthly. No participants used supplements with macroalgae.

## 4 DISCUSSION

In this study, the analyzed average iodine content in the sushi and wakame meal was 231 μg per serving, which corresponded to one tablet of iodine supplement (225 μg) used as reference. The estimated bioavailability of iodine during the first 24 h was 75% from sushi with wakame and 97% from the KI supplement. The bioequivalence analyses also confirmed that the KI supplement had higher estimated bioavailability than the sushi meal, however, with small margins.

The analyzed iodine content in one serving of the sushi and wakame meal (231 μg) corresponded well to the declared iodine content in the KI supplement (225 μg). The iodine content in the sushi and wakame meal did not exceed the tolerable upper intake level (UL) of 600 μg/day for adults used by EFSA (SCF, 2002). Thus, based on these findings, the meal could serve as a dietary iodine source. However, an iodine intake of 231 μg in a single meal is more than adequate compared to the recommended intake of 150 μg/day. Moreover, the amount of iodine present within different species of macroalgae may vary, for example, for nori (*Porphyra* spp.), the iodine content was found to vary from 8 to 100 μg/g (no. of samples = 11) (Duinker et al., 2020). Additionally, factors such as processing, storage conditions, harvest location, and the specific part of the seaweed used can also affect the iodine content (Teas et al., 2004). As a result, a larger sample size would be necessary to draw any general conclusions about the iodine content of these foods.

We found an average iodine content in the pooled wakame samples of 2.3 μg/g wet weight, which was considerably lower than what was found in a study of commercially available products with wakame from three sample sites in Australia, New Zealand, and Japan where the iodine content was 41, 115, and 42 μg/g, respectively (Teas et al., 2004). Similarly, dried wakame was found to contain 104 and 127 μg/g in a British study (Lee et al., 1994). The samples analyzed in our study were products that were already prepared for consumption and had undergone some form of processing. However, there was no information available regarding the specific processing techniques used. Previous findings report that cooking dried wakame reduced the iodine content substantially (Aakre, Doblaug Solli, et al., 2021), which could explain why the iodine content in the analyzed wakame samples used here was lower than for whole wakame products.

The UIC after consumption of the two interventions shows that both groups had higher median UIC in the first 24 h compared to the 24‐ to 48‐h period, indicating that most of the iodine load was excreted within 24 h. This is in line with previous findings reporting that iodine appears in the urine shortly after intake and is cleared within 24–48 h (Nath et al., 1992). Median UIC was substantially different between the intervention groups during the first 24 h, which indicated that the iodine from the KI supplement was excreted in urine to a greater extent than the sushi meal during this period. A British study with a KI and macroalgae supplement (*Ascophyllum nodosum*) found the iodine excretion to peak after 0–2 h in the KI supplement group and after 2–5 h in the seaweed supplement group (Combet et al., 2014), comparable to our findings. In a Greenlandic study measuring UIC and urinary iodine excretion (UIE) after ingestion of a seaweed salad (*Fucus vesiculosus*) and a Japanese seaweed salad without further specification of species, most of the iodine load was excreted during the first 24 h, which is in accordance with our findings (Noahsen et al., 2020). While the KI supplement seemed to give a rapid increase in the UIC, the sushi and wakame meal led to a more moderate initial increase. It has been suggested that the iodine in seaweeds may be “trapped” within the seaweed matrix, bound in organic compounds, and thus less available for absorption (Cherry et al., 2019). A “delayed” iodine excretion was observed in the mentioned Greenlandic study, which proposed that iodine excretion could last up to 36 and perhaps 48 h after seaweed ingestion (Noahsen et al., 2020). In addition to the reduced absorption, some of the iodine may have been excreted in feces, and consequently, less iodine would appear in the urine; this has been demonstrated in an animal trial with rats (Fjære et al., 2022).

The estimated bioavailability in our study showed a higher bioavailability of iodine from the KI supplement (97%) compared to iodine from the sushi and wakame meal (75%) based on the estimates from the first 24 h. In other studies (Andersen et al., 2019; Aquaron et al., 2002; Combet et al., 2014; Miyai et al., 2008; Teas et al., 2007), both higher and lower bioavailability of iodine from macroalgae have been found, ranging from 33% to 100%, however, these studies have investigated different macroalgae species from the current study. Bioavailability of iodine from rockweed (*Ascophyllum nodosum*) and Irish moss (*Chondrus crispus*) was found to be 42% and 50%, respectively, in a Greenlandic study (Andersen et al., 2019), and 33% for a supplement with rockweed in a British study (Combet et al., 2014). Aquaron et al. investigated iodine bioavailability in iodine‐sufficient and ‐insufficient individuals, and found a bioavailability of 101% and 85%, respectively, for *Gracilaria verrucosa* and 90% and 61.5%, respectively, for tangle (*Laminaria hyperborea*) (Aquaron et al., 2002). In a study using seaweed powder from winged kelp (*Alaria esculenta*) in capsules, the bioavailability was found to be 60% (Teas et al., 2007), while a Japanese study examining high iodine doses from Kombu (*Laminaria japonica*) estimated the bioavailability to be 57% and 71%, depending on dose (Miyai et al., 2008). Reasons for the great variability in bioavailability could be not only interspecies differences, differences in methodology used, and study design but also the large variations in iodine content of the interventions ranging from 475 to 70,000 μg of iodine.

Wakame was the component of the sushi meal with the highest iodine content (230 μg/100 g). Chemical species of iodine are suggested to affect the bioavailability of iodine from seaweeds (Hou, 2009). Studies have found that wakame mainly contains iodine in the form of iodide and smaller amounts of iodate (Gamallo‐Lorenzo et al., 2005; Romarís‐Hortas et al., 2013), however, organic forms such as MIT and DIT have also been identified (Hou, 2009). The previously mentioned study by Aquaron et al. found that the bioavailability from MIT was lower (80%) than the bioavailability from inorganic iodine in the form of KI (96%) (Aquaron et al., 2002), which could be an explanation as to why the bioavailability of the sushi meal seemed lower compared to the KI supplement. Additionally, it should be noted that the wakame utilized in this study was subjected to processing, and as a result, the iodine content that remains may be less bioavailable. Nevertheless, there is no existing documentation to confirm this.

The iodine status in the study population may have affected the uptake of iodine (Zimmermann, 2009) as the subjects were mildly iodine deficient according to the WHO criteria (WHO, 2007) with a median UIC of 79 μg/L. The body's mechanism in the presence of iodine deficiency is iodine retention due to increased thyroidal uptake and may have reduced amount of iodine excreted in the urine in this study population (Gowachirapant et al., 2009). None of the participants in this study had thyroid dysfunction. Three individuals were TgAg and TPOAb positive, however, elevated TPOAb is a normal finding in a female population and was reported in 13.9% of females without known thyroid disease in a large Norwegian cohort (Bjoro et al., 2000), where the study population was considered euthyroid.

### 4.1 Strengths and limitations

To the best of our knowledge, this is the first study to assess iodine content in a sushi and wakame meal consisting of two commonly consumed seaweeds and to estimate the bioavailability and bioequivalence in a sushi and wakame meal compared to a reference potassium iodide supplement. The estimates of bioavailability in the present study have several potential limitations. Data on UIE from total urine collections or creatinine excretion were not obtained, therefore an estimated urine volume was used in the calculations. The assessment of bioequivalence showed that the KI supplement was more bioavailable than the sushi; however, for the entire 48‐h period, the lower CI limit of 0.78 is only just outside the limit of 0.8 of bioequivalence. These findings should be interpreted with caution, as the AUC was calculated from spot samples in time intervals, as opposed to 24‐h urine collection and fixed time points (Troja & Deda, 2021). However, 24‐h urine collections were not feasible in this study due to the constraints on the participants.

Variations in iodine intake between the two groups may also have affected our results. Nori and wakame were the macroalgae species included in the sushi meal and were considered the main sources of iodine since the sushi consisted of mainly salmon which has a relatively low content of iodine (6 μg/100 g) (Norwegian Food Safety Authority et al., 2011). Even if six participants did not consume the entire sushi meal, they mostly left behind the nigiri pieces consisting of rice and salmon, which we believe has little contribution to the total iodine intake from the meal. Furthermore, data on total food intake, including goitrogenic foods, during interventions were not collected, which may have influenced our results. However, the participants showed good compliance with the dietary restrictions and the average calculated amount of ingested iodine from the reported deviations was under 10 μg of iodine per day, which means there were few other dietary iodine sources during the interventions. There were some variations in the iodine content between the different batches of the sushi meal, and the iodine content was a bit lower than the declared content of the supplement. We cannot exclude the fact that this has affected our results. Furthermore, we have not analyzed iodine values for the supplement, and cannot account for possible variations in actual versus declared content.

## 5 CONCLUSION

We found that a ready‐to‐eat meal with sushi and wakame including the macroalgae species nori (*Porphyra* spp.) and wakame (*Undaria pinnatifida*) contained 231 μg iodine per serving. The median UIC increased rapidly after intake both for the sushi and wakame meal and the KI supplement, with the highest peak for the supplement. The estimated bioavailability showed that iodine from the sushi and wakame meal (75%) was less available than from the KI supplement (97%). This was supported by the bioequivalence analyses, which demonstrated that the iodine from the KI supplement had higher bioavailability than the sushi and wakame meal, but the differences were minor. Our findings imply that sushi and wakame could be potential iodine sources in the diet, which may be favorable for population groups at risk for iodine deficiency. However, further research is needed to account for the variability of iodine content based on species location and degree of processing in order to ensure safe and predictable products for consumers.

## AUTHOR CONTRIBUTIONS

**Inger Aakre:** Conceptualization (lead); data curation (equal); formal analysis (equal); funding acquisition (equal); investigation (equal); methodology (equal); project administration (equal); writing – original draft (equal). **Inger Beate Tveit:** Data curation (equal); investigation (equal); methodology (equal); writing – review and editing (equal). **Lene Secher Myrmel:** Formal analysis (equal); validation (equal); visualization (equal); writing – review and editing (equal). **Even Fjære:** Methodology (supporting); software (supporting); supervision (supporting); writing – review and editing (equal). **Simon Ballance:** Formal analysis (equal); methodology (equal); software (supporting); writing – review and editing (equal). **Hanne Rosendahl‐Riise:** Conceptualization (equal); data curation (equal); funding acquisition (equal); investigation (equal); project administration (equal); resources (equal); supervision (equal); writing – review and editing (equal).

## FUNDING INFORMATION

This study was financed by the Institute of Marine Research and the University of Bergen. The contribution of Simon Ballance was supported by the Norwegian Seaweed Biorefinery Platform (Research Council of Norway (RCN), grant no 294946/E40).

## CONFLICT OF INTEREST STATEMENT

The authors declare that they do not have any conflict of interest.

## INFORMED CONSENT

Written informed consent was obtained from all study participants. All study personnel have attended the Good Clinical Practice course.

## Supporting information


Appendix S1

Figure S1

Table S1

## Data Availability

Data on urinary iodine concentrations and BE analyses are attached as Supplementary File (Appendix S1).

## References

[fsn33689-bib-0001] Aakre, I. , Doblaug Solli, D. , Wik Markhus, M. , Mæhre, H. K. , Dahl, L. , Henjum, S. , Alexander, J. , Korneliussen, P.‐A. , Madsen, L. , & Kjellevold, M. (2021). Commercially available kelp and seaweed products – Valuable iodine source or risk of excess intake? Food & Nutrition Research, 65. 10.29219/fnr.v65.7584 PMC8035890 33889064

[fsn33689-bib-0002] Aakre, I. , Morseth, M. S. , Dahl, L. , Henjum, S. , Kjellevold, M. , Moe, V. , Smith, L. , & Markhus, M. W. (2021). Iodine status during pregnancy and at 6 weeks, 6, 12 and 18 months post‐partum. Maternal & Child Nutrition, 17(1), e13050. 10.1111/mcn.13050 32602197 PMC7729798

[fsn33689-bib-0003] Abel, M. H. , Caspersen, I. H. , Meltzer, H. M. , Haugen, M. , Brandlistuen, R. E. , Aase, H. , Alexander, J. , Torheim, L. E. , & Brantsæter, A.‐L. (2017). Suboptimal maternal iodine intake is associated with impaired child neurodevelopment at 3 years of age in the Norwegian mother and child cohort study. Journal of Nutrition, 147(7), 1314–1324. 28515161 10.3945/jn.117.250456

[fsn33689-bib-0004] Abel, M. H. , Caspersen, I. H. , Sengpiel, V. , Jacobsson, B. , Meltzer, H. M. , Magnus, P. , Alexander, J. , & Brantsæter, A. L. (2020). Insufficient maternal iodine intake is associated with subfecundity, reduced foetal growth, and adverse pregnancy outcomes in the Norwegian mother, father and child cohort study. BMC Medicine, 18(1), 211. 10.1186/s12916-020-01676-w 32778101 PMC7418397

[fsn33689-bib-0005] Abel, M. H. , Korevaar, T. I. M. , Erlund, I. , Villanger, G. D. , Caspersen, I. H. , Arohonka, P. , Alexander, J. , Meltzer, H. M. , & Brantsæter, A. L. (2018). Iodine intake is associated with thyroid function in mild to moderately iodine deficient pregnant women. Thyroid, 28(10), 1359–1371. 10.1089/thy.2018.0305 30132420 PMC6157349

[fsn33689-bib-0006] Andersen, S. , Noahsen, P. , Rex, K. F. , Florian‐Sørensen, H. C. , & Mulvad, G. (2019). Iodine in edible seaweed, its absorption, dietary use, and relation to iodine nutrition in Arctic people. Journal of Medicinal Food, 22(4), 421–426. 10.1089/jmf.2018.0187 30990756

[fsn33689-bib-0007] Aquaron, R. , Delange, F. , Marchal, P. , Lognoné, V. , & Ninane, L. (2002). Bioavailability of seaweed iodine in human beings. Cellular and Molecular Biology (Noisy‐le‐Grand, France), 48(5), 563–569. 12146713

[fsn33689-bib-0008] Bjoro, T. , Holmen, J. , Krüger, O. , Midthjell, K. , Hunstad, K. , Schreiner, T. , Sandnes, L. , & Brochmann, H. (2000). Prevalence of thyroid disease, thyroid dysfunction and thyroid peroxidase antibodies in a large, unselected population. The health study of Nord‐Trondelag (HUNT). European Journal of Endocrinology of the European Federation of Endocrine Societies, 143(5), 639–647. 10.1530/eje.0.1430639 11078988

[fsn33689-bib-0009] Blikra, M. J. , Henjum, S. , & Aakre, I. (2022). Iodine from brown algae in human nutrition, with an emphasis on bioaccessibility, bioavailability, chemistry, and effects of processing: A systematic review. Comprehensive Reviews in Food Science and Food Safety, 21(2), 1517–1536. 10.1111/1541-4337.12918 35233943

[fsn33689-bib-0010] Brent, G. A. (2012). Mechanisms of thyroid hormone action. Journal of Clinical Investigation, 122(9), 3035–3043. 22945636 10.1172/JCI60047 PMC3433956

[fsn33689-bib-0011] Cherry, P. , O'Hara, C. , Magee, P. J. , McSorley, E. M. , & Allsopp, P. J. (2019). Risks and benefits of consuming edible seaweeds. Nutrition Reviews, 77(5), 307–329. 10.1093/nutrit/nuy066 30840077 PMC6551690

[fsn33689-bib-0012] Combet, E. , Ma, Z. F. , Cousins, F. , Thompson, B. , & Lean, M. E. (2014). Low‐level seaweed supplementation improves iodine status in iodine‐insufficient women. British Journal of Nutrition, 112(5), 753–761. 10.1017/s0007114514001573 25006699

[fsn33689-bib-0013] Dahl, L. , Johansson, L. , Julshamn, K. , & Meltzer, H. M. (2004). The iodine content of Norwegian foods and diets. Public Health Nutrition, 7(4), 569–576. 15153264 10.1079/PHN2003554

[fsn33689-bib-0014] Duinker, A. , Kleppe, M. , Fjære, E. , Biancarosa, I. , Heldal, H. E. , Dahl, L. , & Lunestad, B. T. (2020). Knowledge update on macroalgae food and feed safety‐based on data generated in the period 2014–2019 by the Institute of Marine Research, Norway . Rapport fra havforskningen.

[fsn33689-bib-0015] EFSA , Dujardin, B. , Ferreira de Sousa, R. , & Gómez Ruiz, J. Á. (2023). Dietary exposure to heavy metals and iodine intake via consumption of seaweeds and halophytes in the European population. EFSA Journal, 21(1), e07798. 10.2903/j.efsa.2023.7798 36742462 PMC9887633

[fsn33689-bib-0016] EFSA , EFSA Panel on Food Additives and Nutrient Sources added to Food , Younes, M. , Aggett, P. , Aguilar, F. , Crebelli, R. , Dusemund, B. , Filipič, M. , Frutos, M. J. , Galtier, P. , Gundert‐Remy, U. , Kuhnle, G. G. , Lambré, C. , Leblanc, J.‐C. , Lillegaard, I. T. , Moldeus, P. , Mortensen, A. , Oskarsson, A. , Stankovic, I. , … Gott, D. (2021). Guidance on safety evaluation of sources of nutrients and bioavailability of nutrient from the sources (revision 1). EFSA Journal, 19(3), e06552. 10.2903/j.efsa.2021.6552 33815621 PMC8002907

[fsn33689-bib-0017] Fjære, E. , Poulsen, R. , Duinker, A. , Liaset, B. , Hansen, M. , Madsen, L. , & Myrmel, L. S. (2022). Iodine bioavailability and accumulation of arsenic and cadmium in rats fed sugar kelp (*Saccharina latissima*). Food, 11(24). 10.3390/foods11243943 PMC9777903 36553687

[fsn33689-bib-0018] Gamallo‐Lorenzo, D. , Barciela‐Alonso, M. C. , Moreda‐Piñeiro, A. , Bermejo‐Barrera, A. , & Bermejo‐Barrera, P. (2005). Microwave‐assisted alkaline digestion combined with microwave‐assisted distillation for the determination of iodide and total iodine in edible seaweed by catalytic spectrophotometry. Analytica Chimica Acta, 542(2), 287–295. 10.1016/j.aca.2005.04.002

[fsn33689-bib-0019] Gizak, M. , Gorstein, J. , & Andersson, M. (2017). Epidemiology of iodine deficiency. In E. N. Pearce (Ed.), Iodine deficiency disorders and their elimination (pp. 29–43). Springer International Publishing.

[fsn33689-bib-0020] Gowachirapant, S. , Winichagoon, P. , Wyss, L. , Tong, B. , Baumgartner, J. , Melse‐Boonstra, A. , & Zimmermann, M. B. (2009). Urinary iodine concentrations indicate iodine deficiency in pregnant Thai women but iodine sufficiency in their school‐aged children. Journal of Nutrition, 139(6), 1169–1172. 19403711 10.3945/jn.108.100438

[fsn33689-bib-0021] Groufh‐Jacobsen, S. , Hess, S. Y. , Aakre, I. , Folven Gjengedal, E. L. , Blandhoel Pettersen, K. , & Henjum, S. (2020). Vegans, vegetarians and Pescatarians are at risk of iodine deficiency in Norway. Nutrients, 12(11), 3555. 33233534 10.3390/nu12113555 PMC7699510

[fsn33689-bib-0022] Helse Bergen (Laboratorieklinikken) . (2022a). Analyseoversikten . https://analyseoversikten.no/

[fsn33689-bib-0023] Helse Bergen (Laboratorieklinikken) . (2022b). Analyseoversikten: Tyreoglobulinantistoff . https://analyseoversikten.no/analyser/39

[fsn33689-bib-0024] Henjum, S. , Brantsæter, A. L. , Kurniasari, A. , Dahl, L. , Aadland, E. K. , Gjengedal, E. L. F. , Birkeland, S. , & Aakre, I. (2018). Suboptimal iodine status and low iodine knowledge in young Norwegian women. Nutrients, 10(7), 941. 30037088 10.3390/nu10070941 PMC6073112

[fsn33689-bib-0025] Henjum, S. , Lilleengen, A. M. , Aakre, I. , Dudareva, A. , Gjengedal, E. L. F. , Meltzer, H. M. , & Brantsaeter, A. L. (2017). Suboptimal iodine concentration in breastmilk and inadequate iodine intake among lactating women in Norway. Nutrients, 9(7), 643. 10.3390/nu9070643 28640217 PMC5537763

[fsn33689-bib-0026] Hou, X. (2009). Iodine speciation in foodstuffs, tissues, and environmental samples: Iodine species and analytical method (chapter 15). In V. R. Preedy , G. N. Burrow , & R. R. Watson (Eds.), Comprehensive handbook of iodine: Nutritional, biochemical, pathological and therapeutic aspects. Academic Press.

[fsn33689-bib-0027] Iodine Global Network (IGN) . (2017). Global scorecard of iodine nutrition in 2017 . IGN. https://www.ign.org/cm_data/IGN_Global_Scorecard_AllPop_and_PW_May20171.pdf

[fsn33689-bib-0028] Iodine Global Network (IGN) . (2021). Iodine global network . Annual Report 2020.

[fsn33689-bib-0029] IOM . (2001). Reference intakes for vitamin A, vitamin K, arsenic, boron, chromium, copper, iodine, iron, manganese, molybdenum, nickel, silicon, vanadium and zinc: A report of the panel on micronutrients, subcommittees on upper reference levels of nutrients and interpretation and uses of dietary reference intakes, and the standing committee on the scientific evaluation of dietary reference intakes. National Academy Press.

[fsn33689-bib-0030] Julshamn, K. , Dahl, L. , & Eckhoff, K. (2001). Determination of iodine in seafood by inductively coupled plasma/mass spectrometry. Journal of AOAC International, 84(6), 1976–1983. 11767171

[fsn33689-bib-0031] Katagiri, R. , Yuan, X. , Kobayashi, S. , & Sasaki, S. (2017). Effect of excess iodine intake on thyroid diseases in different populations: A systematic review and meta‐analyses including observational studies. PLoS One, 12(3), e0173722. 28282437 10.1371/journal.pone.0173722 PMC5345857

[fsn33689-bib-0032] Laurberg, P. , Pedersen, I. , Carlé, A. , Andersen, S. , Knudsen, N. , Ovesen, L. , & Rasmussen, L. B. (2009). The U‐shaped curve of iodine intake and thyroid disorders. In V. R. Preedy , G. N. Burrow , & R. Watson (Eds.), Comprehensive handbook on iodine: Nutritional, endocrine and pathological aspects (pp. 449–455). Elsevier.

[fsn33689-bib-0033] Lee, S. M. , Lewis, J. , Buss, D. H. , Holcombe, G. D. , & Lawrance, P. R. (1994). Iodine in British foods and diets. British Journal of Nutrition, 72(3), 435–446. 10.1079/BJN19940045 7947658

[fsn33689-bib-0034] Mæhre, H. K. , Malde, M. K. , Eilertsen, K.‐E. , & Elvevoll, E. O. (2014). Characterization of protein, lipid and mineral contents in common Norwegian seaweeds and evaluation of their potential as food and feed. Journal of the Science of Food and Agriculture, 94(15), 3281–3290. 10.1002/jsfa.6681 24700148

[fsn33689-bib-0035] Mehra, A. , Saikat, S. Q. , & Carter, J. E. (2014). Bioavailability of iodine in the UK‐Peak District environment and its human bioaccessibility: An assessment of the causes of historical goitre in this area. Environmental Monitoring and Assessment, 186(2), 987–999. 24407919 10.1007/s10661-013-3433-7

[fsn33689-bib-0036] Miyai, K. , Tokushige, T. , & Kondo, M. (2008). Suppression of thyroid function during ingestion of seaweed “kombu” (*Laminaria japonoca*) in normal Japanese adults. Endocrine Journal, 55(6), 1103–1108. 10.1507/endocrj.k08e-125 18689954

[fsn33689-bib-0037] Nath, S. , Moinier, B. , Thuillier, F. , Rongier, M. , & Desjeux, J. (1992). Urinary excretion of iodide and fluoride from supplemented food grade salt. International Journal for Vitamin and Nutrition Research, 62(1), 66–72. 1587711

[fsn33689-bib-0038] Nerhus, I. , Wik Markhus, M. , Nilsen, B. , Øyen, J. , Maage, A. , Ødegård, E. , Kolden Midtbø, L. , Frantzen, S. , Kögel, T. , Eide Graff, I. , Lie, Ø. , Dahl, L. , & Kjellevold, M. (2018). Iodine content of six fish species, Norwegian dairy products and hen's egg. Food & Nutrition Research, 62. 10.29219/fnr.v62.1291 PMC5971469 29853825

[fsn33689-bib-0039] Noahsen, P. , Kleist, I. , Larsen, H. M. , & Andersen, S. (2020). Intake of seaweed as part of a single sushi meal, iodine excretion and thyroid function in euthyroid subjects: A randomized dinner study. Journal of Endocrinological Investigation, 43(4), 431–438. 10.1007/s40618-019-01122-6 31571150

[fsn33689-bib-0040] Norwegian Directorate of Health, & Norwegian Food safety Authority . (2022). Kostholdplanleggeren . https://www.kostholdsplanleggeren.no/

[fsn33689-bib-0041] Norwegian Food Safety Authority . (2022). Norwegian food composition database . www.matvaretabellen.no

[fsn33689-bib-0042] Romarís‐Hortas, V. , Bermejo‐Barrera, P. , & Moreda‐Piñeiro, A. (2013). Ultrasound‐assisted enzymatic hydrolysis for iodinated amino acid extraction from edible seaweed before reversed‐phase high performance liquid chromatography‐inductively coupled plasma‐mass spectrometry. Journal of Chromatography A, 1309, 33–40. 10.1016/j.chroma.2013.08.022 23972456

[fsn33689-bib-0043] Rosendahl‐Riise, H. , Aksnes, S. , Sabir, Z. , Ulleberg, E. K. , Myklebust‐Hansen, T. , & Aakre, I. (2023). Comparison of a digital iodine‐specific dietary screener with 24‐hour recall and urinary iodine concentration. Journal of Nutritional Science, 12, e90, 10.1017/jns.2023.74 37592931 PMC10427488

[fsn33689-bib-0044] Scientific Committee on Food (SCF) . (2002). Opinion of the Scientific Committee on Food on the tolerable upper intake level of iodine. Brussels: European Commission.

[fsn33689-bib-0045] Teas, J. , Braverman, L. E. , Kurzer, M. S. , Pino, S. , Hurley, T. G. , & Hebert, J. R. (2007). Seaweed and soy: Companion foods in Asian cuisine and their effects on thyroid function in American women. Journal of Medicinal Food, 10(1), 90–100. 10.1089/jmf.2005.056 17472472

[fsn33689-bib-0046] Teas, J. , Pino, S. , Critchley, A. , & Braverman, L. E. (2004). Variability of iodine content in common commercially available edible seaweeds. Thyroid, 14(10), 836–841. 15588380 10.1089/thy.2004.14.836

[fsn33689-bib-0047] The Norwegian Directorate of Health . (2021). Utviklingen i norsk kosthold . https://www.helsedirektoratet.no/rapporter/utviklingen‐i‐norsk‐kosthold

[fsn33689-bib-0048] Troja, E. , & Deda, L. (2021). Assessment of bioequivalence using urinary excretion data. Journal of Bioequivalence & Bioavailability, 13(4), 443.

[fsn33689-bib-0049] WHO . (2007). Assessment of iodine deficiency disorders and monitoring their elimination. A guide for programme managers. World Health Organization.

[fsn33689-bib-0050] Zimmermann, B. M. (2012). Assessment of iodine nutrition in populations: Past, present, and future. Nutrition Reviews, 70(10), 553–570. 23035804 10.1111/j.1753-4887.2012.00528.x

[fsn33689-bib-0051] Zimmermann, M. B. (2009). Iodine deficiency. Endocrine Reviews, 30(4), 376–408. 19460960 10.1210/er.2009-0011

