# Correction to “Bioavailability of Iodine From a Meal Consisting of Sushi and a Wakame Seaweed Salad—A Randomized Crossover Trial”

**DOI:** 10.1002/fsn3.70275

**Published:** 2025-05-13

Aakre, I., Tveit, I. B., Myrmel, L. S., Fjære, E., Ballance, S., & Rosendahl‐Riise, H. (2023). Bioavailability of iodine from a meal consisting of sushi and a wakame seaweed salad—A randomized crossover trial. Food Science & Nutrition, 11, 7707–7717. https://doi.org/10.1002/fsn3.3689

In the originally published article, Hanne Rosendahl‐Riise was incorrectly spelled as Hanne Rosendal‐Riise.

The original article has been corrected accordingly.

We apologize for this error.

