# Supplementary material for: Bioavailability of iodine from a meal consisting of sushi and a wakame seaweed salad—A randomized crossover trial
Source: Food Sci Nutr. 2023 Sep 24;11(12):7707–17. doi: 10.1002/fsn3.3689 (PMC10724604; doi:10.1002/fsn3.3689)
Supplement: Supplementary file 1 — Appendix S1 [file FSN3-11-7707-s003.pdf]

AUC 0-48 hours, Subject 18 and 22 removed.

```
Analysis of Variance (log scale)
              Sum Sq Df Mean Sq F value    Pr(>F)
SUBJECT        8.1695 19 0.42997  6.5694 0.0001004 ***
GROUP          0.0177  1 0.01772  0.0391 0.8454026
SUBJECT(GROUP) 8.1518 18 0.45288  6.9193 7.595e-05 ***
PERIOD         0.0042  1 0.00416  0.0635 0.8039084
SUSHI MEAL     0.1046  1 0.10459  1.5980 0.2223111
ERROR          1.1781 18 0.06545
TOTAL          9.4522 39
---
Signif. codes:  0 ‘***’ 0.001 ‘**’ 0.01 ‘*’ 0.05 ‘.’ 0.1 ‘ ’ 1

Between and Within Subject Variability
                              Between Subject Within Subject
Variance Estimate                  0.1937128     0.06545106
Coefficient of Variation, CV(%)   46.2328457    26.00778524

Least Square Means (geometric mean)
              Reference (R)   Sushi meal (T)
Geometric Means 3945.906        3554.803

90% Confidence Interval of Geometric Mean Ratio (T/R)`
              Lower Limit Point Estimate Upper Limit
90% CI for Ratio    0.780703       0.900884    1.039566
```

Cmax 0-48 hours

```
Analysis of Variance (log scale)
              Sum Sq Df Mean Sq F value  Pr(>F)
SUBJECT        6.0007 19 0.31583  1.9644 0.07919 .
GROUP          0.0020  1 0.00200  0.0060 0.93916
SUBJECT(GROUP) 5.9987 18 0.33326  2.0728 0.06568 .
PERIOD         0.0723  1 0.07230  0.4497 0.51099
SUSHI MEAL     0.5257  1 0.52572  3.2698 0.08730 .
ERROR          2.8940 18 0.16078
TOTAL          9.4365 39
---
Signif. codes:  0 ‘***’ 0.001 ‘**’ 0.01 ‘*’ 0.05 ‘.’ 0.1 ‘ ’ 1

Between and Within Subject Variability
                              Between Subject Within Subject
Variance Estimate                  0.0862425      0.1607786
Coefficient of Variation, CV(%)   30.0117700     41.7642123

Least Square Means (geometric mean)
                    Reference (R)   Sushi meal (T)
Geometric Means        214.7223        169.9208

90% Confidence Interval of Geometric Mean Ratio (T/R)
              Lower Limit Point Estimate Upper Limit
90% CI for Ratio   0.6322795      0.7913515   0.9904436
```

Day 1 AUC

Analysis of Variance (log scale)

```
                Sum Sq Df Mean Sq F value   Pr(>F)
SUBJECT          7.6754 19 0.40397  3.1949 0.008555 **
GROUP            0.0046  1 0.00456  0.0107 0.918748
SUBJECT(GROUP)  7.6709 18 0.42616  3.3704 0.006742 **
PERIOD           0.0001  1 0.00011  0.0008 0.977149
SUSHI MEAL       0.5366  1 0.53663  4.2441 0.054145 .
ERROR            2.2759 18 0.12644
TOTAL           10.5073 39
---
Signif. codes:  0 '***' 0.001 '**' 0.01 '*' 0.05 '.' 0.1 ' ' 1
```

Between and Within Subject Variability

```
                              Between Subject Within Subject
Variance Estimate                  0.1498599      0.1264404
Coefficient of Variation, CV(%)   40.2083936     36.7126498
```

Least Square Means (geometric mean)

```
                  Reference (R)  Sushi meal (T)
Geometric Means       2832.134       2235.805
```

90% Confidence Interval of Geometric Mean Ratio (T/R)

```
              Lower Limit Point Estimate Upper Limit
90% CI for Ratio    0.646981      0.7894415   0.9632709
```

Day 1 Cmax

Analysis of Variance (log scale)

```
               Sum Sq Df Mean Sq F value  Pr(>F)
SUBJECT         6.0802 19 0.32001  1.9477 0.08181 .
GROUP           0.0013  1 0.00134  0.0040 0.95053
SUBJECT(GROUP) 6.0788 18 0.33771  2.0554 0.06789 .
PERIOD          0.0388  1 0.03880  0.2361 0.63288
SUSHI MEAL      0.5777  1 0.57771  3.5161 0.07709 .
ERROR           2.9575 18 0.16430
TOTAL           9.6175 39
---
Signif. codes:  0 '***' 0.001 '**' 0.01 '*' 0.05 '.' 0.1 ' ' 1
```

Between and Within Subject Variability

```
                              Between Subject Within Subject
Variance Estimate                  0.0867045      0.1643044
Coefficient of Variation, CV(%)   30.0955753     42.2578975
```

Least Square Means (geometric mean)

```
                  Reference (R)  Sushi meal (T)
Geometric Means       214.9379  168.1803
```

90% Confidence Interval of Geometric Mean Ratio (T/R)

```
              Lower Limit Point Estimate Upper Limit
90% CI for Ratio   0.6236472        0.78246   0.9817147
```

Day 2 AUC

Analysis of Variance (log scale)

```
                Sum Sq Df Mean Sq F value    Pr(>F)
SUBJECT        12.1400 19 0.63895  6.9151 7.021e-05 ***
```

```
GROUP           0.0422  1 0.04223  0.0628   0.80492
SUBJECT(GROUP) 12.0977 18 0.67210  7.2739 5.348e-05 ***
PERIOD          0.0002  1 0.00015  0.0017   0.96796
SUSHI MEAL      0.3667  1 0.36665  3.9682   0.06176 .
ERROR           1.6632 18 0.09240
TOTAL          14.1884 39
---
Signif. codes:  0 ‘***’ 0.001 ‘**’ 0.01 ‘*’ 0.05 ‘.’ 0.1 ‘ ’ 1
```

Between and Within Subject Variability

	Between Subject	Within Subject
Variance Estimate	0.2898491	0.09239886
Coefficient of Variation, CV(%)	57.9849783	31.11305212

Least Square Means (geometric mean)

	Reference (R)	Sushi meal (T)
Geometric Means	1016.224	1235.56

90% Confidence Interval of Geometric Mean Ratio (T/R)`

	Lower Limit	Point Estimate	Upper Limit
90% CI for Ratio	1.02563	1.215834	1.441311

CMax day 2

Analysis of Variance (log scale)

```
               Sum Sq Df Mean Sq F value    Pr(>F)
SUBJECT        8.7062 19 0.45822  9.0678 9.882e-06 ***
GROUP          0.1069  1 0.10694  0.2239    0.6418
SUBJECT(GROUP) 8.5992 18 0.47773  9.4540 7.954e-06 ***
PERIOD         0.2082  1 0.20824  4.1209    0.0574 .
SUSHI MEAL     0.1002  1 0.10023  1.9834    0.1761
ERROR          0.9096 18 0.05053
TOTAL          9.9973 39
---
Signif. codes:  0 ‘***’ 0.001 ‘**’ 0.01 ‘*’ 0.05 ‘.’ 0.1 ‘ ’ 1
```

Between and Within Subject Variability

	Between Subject	Within Subject
Variance Estimate	0.2136009	0.05053279
Coefficient of Variation, CV(%)	48.7984029	22.76649986

Least Square Means (geometric mean)

	Reference (R)	Suschi meal (T)
Geometric Means	91.20563	101.0176

90% Confidence Interval of Geometric Mean Ratio (T/R)

	Lower Limit	Point Estimate	Upper Limit
90% CI for Ratio	0.9766446	1.10758	1.25607
