# Supplementary material for: Bioavailability of iodine from a meal consisting of sushi and a wakame seaweed salad—A randomized crossover trial
Source: Food Sci Nutr. 2023 Sep 24;11(12):7707–17. doi: 10.1002/fsn3.3689 (PMC10724604; doi:10.1002/fsn3.3689)
Supplement: Supplementary file 2 — Figure S1 [file FSN3-11-7707-s002.pdf]

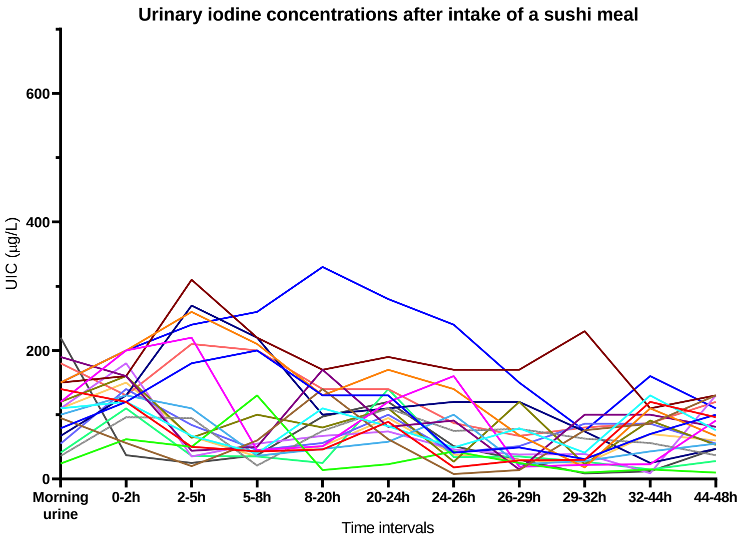
Urinary iodine concentrations after intake of a sushi meal
UIC (µg/L)
0
200
400
600
Morning urine
0-2h
2-5h
5-8h
8-20h
20-24h
24-26h
26-29h
29-32h
32-44h
44-48h
Time intervals

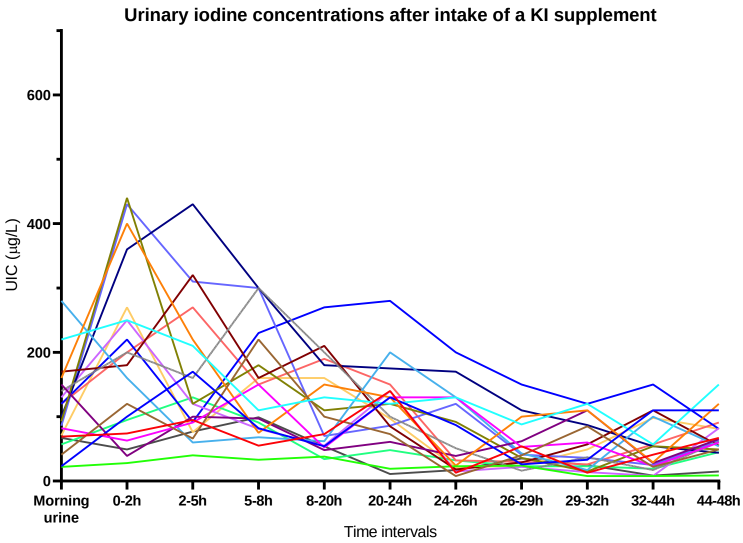
Urinary iodine concentrations after intake of a KI supplement
UIC (µg/L)
0
200
400
600
Morning urine
0-2h
2-5h
5-8h
8-20h
20-24h
24-26h
26-29h
29-32h
32-44h
44-48h
Time intervals
