# Supplementary material for: Bioavailability of iodine from a meal consisting of sushi and a wakame seaweed salad—A randomized crossover trial
Source: Food Sci Nutr. 2023 Sep 24;11(12):7707–17. doi: 10.1002/fsn3.3689 (PMC10724604; doi:10.1002/fsn3.3689)
Supplement: Supplementary file 3 — Table S1 [file FSN3-11-7707-s001.docx]

**Supplementary**

**Table S1.** Estimated iodine intake (μg) from dietary restriction deviations during interventions

| Study arms | | First intervention | | Second intervention | | |
| --- | --- | --- | --- | --- | --- | --- |
|  | **Mean (μg iodine)** | | **SD** | **Mean (μg iodine)** | | **SD** |
| KI – SW (n=12) | | 6.6 | 11.5 | 7.2 | 12.5 | |
| SW – KI (n=8) | | 8.4 | 7.5 | 6.2 | 9.1 | |

KI, Potassium iodide supplement

SW, Sushi and wakame salad

**Figure S1**. Urinary iodine concentrations for each participant over 48 hours after ingestion of a sushi meal with wakame (left) and a KI supplement (right) (n=20).
